# Comparative genomics of thermosynechococcaceae and thermostichaceae: insights into codon usage bias

**DOI:** 10.3389/abp.2024.13825

**Published:** 2025-01-08

**Authors:** Qiao-Hui Mou, Zhe Hu, Jing Zhang, Maurycy Daroch, Jie Tang

**Affiliations:** ^1^ School of Food and Bioengineering, Chengdu University, Chengdu, China; ^2^ Food Safety Detection Key Laboratory of Sichuan, Technical Center of Chengdu Customs, Chengdu, China; ^3^ School of Environment and Energy, Peking University Shenzhen Graduate School, Shenzhen, China

**Keywords:** thermosynechococcaceae, thermostichaceae, codon usage, evolution, natural selection

## Abstract

Members of the families Thermosynechococcaceae and Thermostichaceae are well-known unicellular thermophilic cyanobacteria and a non-thermophilic genus *Pseudocalidococcus* was newly classified into the former. Analysis of the codon usage bias (CUB) of cyanobacterial species inhabiting different thermal and non-thermal niches will benefit the understanding of their genetic and evolutionary characteristics. Herein, the CUB and codon context patterns of protein-coding genes were systematically analyzed and compared between members of the two families. Overall, the nucleotide composition and CUB indices were found to differ between thermophiles and non-thermophiles. The thermophiles showed a higher G/C content in the codon base composition and tended to end with G/C compared to the non-thermophiles. Correlation analysis indicated significant associations between codon base composition and CUB indices. The results of the effective number of codons, parity-rule 2, neutral and correspondence analyses indicated that mutational pressure and natural selection primarily account for CUB in these cyanobacterial species, but the primary driving forces exhibit variation among genera. Moreover, the optimal codons identified based on relative synonymous codon usage values were found to differ among genera and even within genera. In addition, codon context pattern analysis revealed the specificity of the sequence context of start and stop codons among genera. Intriguingly, the clustering of codon context patterns appeared to be more related to thermotolerance than to phylogenomic relationships. In conclusion, this study facilitates the understanding of the characteristics and sources of variation of CUB and the evolution of the surveyed cyanobacterial clades with different thermotolerance and provides insights into their adaptation to different environments.

## Introduction

Codons (64 nucleotide triplets) have been deciphered as the genetic code of the DNA in organisms (Koonin and Novozhilov, 2009). The standard amino acids are encoded by sixty-one codons, and the remaining three codons represent translation termination signals. An amino acid can be encoded by several synonymous codons due to the degeneracy of the genetic code (Yu et al., 2021), with the exception of tryptophan and methionine. Nevertheless, it is known that certain synonymous codons are preferentially used to encode an amino acid (Yang et al., 2021). Codon usage bias (CUB) is used to describe such variable usage frequencies of synonymous codons and has been widely observed across species in microbial organisms (Arella et al., 2021). Codon usage (CU) bias exists among specific genes and genomes. Although synonymous or silent mutations have no functional consequences, they cause synonymous codon variations in genomes during evolution. Therefore, CUB happens because of biased mutational patterns, whereby some codons may be more susceptible to mutation than others and sustained by selection (Bailey et al., 2021; Parvathy et al., 2022). In addition, CUB could be influenced by GC heterogeneity and GC-biased gene conversion (gBGC) (Dilucca et al., 2021). Thus, the evolution of synonymous CU represents a balance between genetic drift, mutation, and natural selection that results in the translational efficiency of genes, which could be considerably responsible for genome evolution (Mazumdar et al., 2017; Yao et al., 2019). The mutational mechanism speculates that codon bias is caused by nucleotide biases generated by point mutations, or in the rates or repairs of point mutations and is responsible for the interspecific variation of CU (Wang et al., 2011; Gómez et al., 2020). Natural selection and evolution promote or suppress synonymous mutations that affect the adaptability of an organism, leading to CU variation across a genome or a gene (Krasovec and Filatov, 2019; Parvathy et al., 2022).

Codon biases are considered to be associated with extensive broad processes. Codon bias can determine transcription levels by influencing chromatin structure and mRNA folding (Kokate et al., 2021). It can also affect translation efficiency by regulating the elongation rate of translation (Liu, 2020). Thus, codon bias can result from genomic adaptation to transcription and/or translation or adaptation alone (Biswas et al., 2019). In light of similar patterns of CU among closely related organisms, codon bias analysis can unravel horizontal gene transfer (HGT) and evolutionary relationships between organisms (Parvathy et al., 2022). Moreover, the majority of proteins with high expression levels are encoded by genes composed of optimal codons (Frumkin et al., 2018). Thus, codon optimization can be applied to improve heterologous gene expression in transgenic studies.

Members of the family Thermosynechococcaceae and the family Thermostichaceae are known as unicellular thermophilic *Synechococcus*-like cyanobacterial strains. They are essential components of high-temperature microbial mats and major producers of geothermal habitats and have been discovered to be cosmopolitan in diverse thermal environments (Tang et al., 2018a; Tang et al., 2018b; Alcorta et al., 2020; Prondzinsky et al., 2021). The family Thermosynechococcaceae represents a distinctive cyanobacterial clade characterized by solitary cells and very rarely short filaments (composed of 2–4 connected cells) (Komárek et al., 2020). Initially, *Thermosynechococcus* was the only described genus within this family (Katoh et al., 2001). Recently, another thermophilic genus, *Parathermosynechococcus* (Tang et al., 2024b) which was previously regarded as a member of *Thermosynechococcus,* has been identified. More intriguingly, several *Synechococcus*-like strains recovered from freshwater lakes were newly classified into the family as the genus *Pseudocalidococcus* (Luz et al., 2023). Such habitat distribution and genus etymology indicate that *Pseudocalidococcus* is not thermophilic. In contrast to the recently diverged Thermosynechococcaceae clade (Shih et al., 2017), Thermostichaceae is regarded as early-branching/early-divergent *Synechococcus*, closer to the root of the cyanobacterial phylogram, typically *Gloeobacter* (Blank and Sanchez Baracaldo, 2010). This clade represented by the single genus *Thermostichus* is the most thermophilic cyanobacteria described to date (Kees et al., 2022).

The thermophilic cyanobacteria of the two families naturally thrive in selective environments characterized by extremes of pH, temperature, etc., (Kees et al., 2022; Pierpont, 2022; Tang et al., 2022c). It is well known that the association of these factors is deleterious to the majority of organisms and could be hypothesized to be the main driving force to shape these evolutionarily adapted thermophilic cyanobacteria. Although previous studies have been carried out on CU in certain cyanobacterial genomes (Prabha et al., 2012; Yu et al., 2012; Xu et al., 2013; Prabha et al., 2017), a comprehensive genome-wide profile representing the taxonomic group of thermophilic cyanobacteria is still lacking in understanding the genetic characteristics of codon bias in thermophilic species. The analysis of CU in genomes of cyanobacterial species inhabiting different thermal and non-thermal niches will thus provide insights into their adaptation to distinct environments and evolutionary diversification. Therefore, the investigation of the base bias of members from the families Thermosynechococcaceae and Thermostichaceae will benefit the understanding of their genetic and evolutionary characteristics.

Herein, the CUB and codon context patterns of genomic CDSs were systematically characterized and compared among members of the families Thermosynechococcaceae and Thermostichaceae. These results revealed features and sources of variation of CUB and phylogenetic relationships concerning their lineages. In addition, CU analysis may be useful in applications of improving gene expression efficiency in genetic transformation research through codon optimization.

## Materials and methods

### Dataset construction

Following the taxonomy of the families Thermosynechococcaceae and Thermostichaceae in the literature (Komárek et al., 2020; Luz et al., 2023; Tang et al., 2024b), representatives were first selected based on genome availability and genome quality (near completeness, >95%; low contamination, <2%; number of ambiguous bases) as previously described (Tang et al., 2022b). Furthermore, genome dereplication was performed using dRep v2.3.2 (Olm et al., 2017) with default settings to eliminate overrepresented species. Only one representative from each species was retained based on the score generated by dRep. Finally, a dataset of 19 genomes was established (Table 1). The genomes of the surveyed cyanobacterial species were retrieved from the NCBI genome database. To avoid gene annotation bias, all the genomes were annotated using the RAST annotation system (Brettin et al., 2015).

**TABLE 1:** Details and genomic features of the studied cyanobacterial species.

Family	Genus/species	Habitats	Genome size (Mb)	GC content (%)	Number of coding genes	Accession number
Thermosynechococcaceae	*Pseudocalidococcus azoricus* BACA0444	Freshwater lake	3.5	48.5	3,642	ASM3172905
	*Pseudocalidococcus* sp. PCC 6312	Freshwater lake	3.7	48.5	3,979	CP003558
	*Parathermosynechococcus sichuanensis* PCC 6715	Hot spring, 53°C, pH 8.4	2.7	53.5	2,814	CP018092
	*Thermosynechococcus nakabusensis* NK55	Hot spring, 52°C–60°C, pH 8.5–9	2.5	54.0	2,540	CP006735
	*Thermosynechococcus sichuanensis* E542	Hot spring, 67°C, pH 8.0	2.7	53.5	2,574	CP032152
	*Thermosynechococcus taiwanensis* CL-1	Hot spring, 62°C, pH 9.3	2.6	53.5	2,604	CP040671
	*Thermosynechococcus taiwanensis* TA-1	Hot spring, 50°C, pH 7-9	2.7	53.5	2,616	CP070960
	*Thermosynechococcus vestitus* BP-1	Hot spring, 55°C, pH 7.5	2.6	54.0	2,609	BA000039
	*Thermosynechococcus* sp. HN-54	Hot spring	2.7	53.0	2,657	CP098039
	*Thermosynechococcus* sp. KatS	Hot spring, 52°C, pH 7.2	2.5	54.0	2,480	AP024678
	*Thermosynechococcus* sp. M55	Hot spring, 55°C, pH 7.9	2.4	54.0	2,438	ASM1529591
	*Thermosynechococcus* sp. PP45	Hot spring	2.7	53.5	2,656	CP130344
	*Thermosynechococcus* sp. Uc	Hot spring	2.3	52.5	2,445	ASM2933599
Thermostichaceae	*Thermostichus* sp. JA-2-3Ba	Hot spring, 56°C	3.0	58.5	3,106	CP000240
	*Thermostichus* sp. JA-3-3Ab	Hot spring, 61.5°C	2.9	60.0	3,010	CP000239
	*Thermostichus* sp. M44	Hot spring, 44.5°C	2.9	58.0	2,991	ASM1529598
	*Thermostichus* sp. MAXBIN	NA	3.0	54.5	3,049	GCA_937936735
	Thermostichus sp. Nb3U1	Hot spring, 49.9°C	3.5	55.0	3,482	ASM2153383
	*Thermostichus vulcanus* Rupite	Hot spring	3.7	55.0	3,828	ASM2284890

NA, not available.

### CU and CUB indices

A total of 10 indices were determined to evaluate the CU and CUB in each genome used. Excluding Met, Trp, and termination codons, the frequency of guanine and cytosine at the third synonymous position was evaluated by the GC3s parameter (Huo et al., 2021). Hydrophobic (positive values) and hydrophilic (negative values) proteins were represented by general average hydropathicity (GRAVY) values ranging from −2 to 2, respectively. The frequency of aromatic amino acids was represented by the aromaticity (AROMO) value. The RSCU (Relative Synonymous Codon Usage) values > 1 and <1 suggested positive codon bias and negative codon bias, respectively, whereas a random or equal CU was suggested when the RSCU value was equal to 1 (Franzo et al., 2021). The codon adaptation index (CAI) refers to the relative adaptation of the CU of a gene to the CU of highly expressed genes. CAI values range between 0 and 1.0, with higher values representing greater codon usage bias (Sharp and Li, 1987). The Codon Bias Index (CBI) quantifies the degree to which preferred codons are utilized in genetic sequences (Bahiri-Elitzur and Tuller, 2021). The frequency of optimal codons (FOP) was evaluated as described (Li et al., 2023). The values of ENC (Effective Number of Codons) estimated the codon bias for each gene, varying from 20 to 61. Only one codon was used for the amino acid in genes when the ENC value was equal to 20, while 61 suggested no CU preference (Sharp and Li, 1987). In the case of an ENC value <36, the gene was susceptible to strong CU preference (Novembre, 2002). All the indices mentioned above were calculated using CodonW1.4.2^1^ and the CAIcal server (Puigbò et al., 2008).

### Analysis of CUB sources

The neutrality plot was illustrated using GC12, which represents the average ratio of GC content in the first (GC1) and second positions (GC2) of the codons, and GC3, which represents the GC content in the third position. If a statistically strong correlation was found between GC12 and GC3, mutational pressure was the dominant driving force. Conversely, natural selection is the major driving force (Parvathy et al., 2022).

The ENC vs. GC3s was plotted to estimate whether the CU of a specific gene is influenced only by mutation or also by other factors, e.g., natural selection. Further, the expected curve on the ENC-GC3s plot was calculated using the equation below. If the corresponding point was distributed around the expected curve, mutational pressure was concluded to be the independent force in the formation of codon bias. Otherwise, certain other factors, e.g., natural selection, played a key role in the formation of codon bias (Sueoka, 1988).

$${ENC}_{\exp}=2+\text{GC}3s+\frac{29}{{GC3}_{S}^{2}+{\left(1-GC3s\right)}^{2}}$$

The ENC_Ratio_ index was further calculated to represent the variations between the expected value and the actual value of ENC using the following equation.

$${ENC}_{Ratio}=\frac{{ENC}_{\exp}-{ENC}_{obs}}{{ENC}_{\exp}}$$

The frequency of each nucleotide at the third position of the codon (A3, U3, G3, and C3) was collected to plot the Parity Rule 2 bias (PR2-Bias) using the following data: A3/(A3 + U3) vs. G3/(G3 + C3) (Wang et al., 2016).

### Correspondence analysis of CU

The multivariate statistical analysis of CU patterns was determined by correspondence analysis (COA). Excluding the unique Met and Trp codons from the 61 codons, the genes comprise 59 sense codons. All the genes were placed into a 59-dimensional hyperspace in the plot. The method indicates the prevailing trend of CU variation in the CDS of the genomes and allocates codons along the axis based on the RSCU value.

### Optimal codon identification

According to the CAI values, the top and bottom 5% of all the surveyed genes were refined to generate a high and low-expression gene dataset for each genome. The D-value between the mean RSCU for each codon of the two datasets (ΔRSCU) was calculated to define codons with ΔRSCU greater than 0.08 as high expression. Codons with RSCU values >1 were considered high-frequency codons. The optimal codon was defined as a codon with ΔRSCU >0.08 and RSCU >1.

### Codon context analysis

All adjacent codon pairs were quantified and subjected to statistical analysis using the residual analysis tool in Anaconda V2.0 software (Moura et al., 2007). The clustering of codon context patterns was depicted using Anaconda.

### Phylogenetic analysis

The phylogenetic inference of the surveyed cyanobacterial species was constructed and further compared based on CU and sequences of single-copy bacterial genes respectively. The hierarchical clustering based on the RSCU values was performed using SPSS v19.0 software. The phylogenetic relationship was reconstructed based on the concatenated sequences of the 120 single-copy genes generated from the GTDB-tk analysis (Chaumeil et al., 2020). Multisequence alignment and ML phylogenetic inference were conducted using MAFFT v7.453 (Katoh and Standley, 2013) and IQ-TREE v2.1.3 (Minh et al., 2020), respectively. Model selection and parameter setting in IQ-TREE and bootstrap analysis were carried out as described (Tang et al., 2022a).

## Results

### Nucleotide composition

Detailed information regarding genome characteristics and ecology of cyanobacterial species from the families Thermosynechococcaceae and Thermostichaceae is summarized in Table 1. The genomes studied showed distinct base compositions among the four cyanobacterial genera, while intragenic variation was limited (Table 2). The non-thermophilic *Pseudocalidococcus* had the lowest contents of GC1, GC2 and GC3, while *Thermostichus* had the highest. *Parathermosynechococcus* and *Thermosynechococcus* showed a similar base composition. The GC3 (48.11%) of *Pseudocalidococcus* was found to be significantly lower (54.73%–64.06%) than that of the other three thermophilic genera, implying a different preference for the third position of codons. All the genomes exhibited an uneven base composition, suggesting that the codons of the genes in these genomes tend to start with and/or end in G/C. The average GC content was found to be 48.50% for *Pseudocalidococcus*, 53.50% for *Parathermosynechococcus*, 53.55% for *Thermosynechococcus*, and 56.83% for *Thermostichus*. This result indicates a different overall preference for codons containing G and C among the four genera, particularly between thermophilic species and non-thermophilic species.

**TABLE 2:** Summary of the CU and CUB indices of the studied cyanobacterial genomes.

Species	CAI	CBI	FOP	GRAVY	AROMO	ENC	GC1 (%)	GC2 (%)	GC3 (%)	GC3s(%)
*Pseudocalidococcus* BACA0444	0.20	−0.05	0.38	−0.06	0.08	49.23	56.04	40.30	48.11	0.46
*Pseudocalidococcus* PCC 6312	0.20	−0.04	0.38	−0.09	0.08	49.52	56.04	40.30	48.11	0.46
*Parathermosynechococcus* PCC 6715	0.22	0.02	0.42	−0.03	0.08	49.74	60.92	42.84	56.73	0.55
*Thermosynechococcus* NK55	0.22	0.01	0.41	−0.03	0.08	48.95	61.66	42.52	57.26	0.56
*Thermosynechococcus* E542	0.22	0.00	0.41	−0.04	0.08	48.52	61.58	42.17	56.61	0.55
*Thermosynechococcus* CL-1	0.22	0.00	0.41	−0.04	0.08	48.37	61.54	42.11	57.13	0.56
*Thermosynechococcus* TA-1	0.22	0.00	0.41	−0.04	0.08	48.35	61.55	42.18	57.28	0.56
*Thermosynechococcus* BP-1	0.21	0.01	0.41	−0.05	0.08	49.07	61.56	42.69	57.30	0.56
*Thermosynechococcus* HN-54	0.21	0.00	0.40	−0.04	0.08	48.92	61.13	42.05	56.38	0.55
*Thermosynechococcus* KatS	0.22	0.01	0.41	−0.03	0.08	48.57	62.12	42.79	58.22	0.57
*Thermosynechococcus* M55	0.22	0.00	0.41	−0.03	0.08	48.47	61.79	42.38	57.32	0.56
*Thermosynechococcus* PP45	0.22	0.00	0.41	−0.05	0.08	48.40	61.41	42.11	56.98	0.55
*Thermosynechococcus* Uc	0.21	0.00	0.40	−0.02	0.08	49.91	61.05	42.22	54.40	0.53
*Thermostichus* sp. JA-2-3Ba	0.23	0.11	0.47	−0.11	0.08	46.19	62.26	44.34	67.03	0.66
*Thermostichus* sp. JA-3-3Ab	0.24	0.13	0.48	−0.10	0.08	44.55	63.55	44.57	70.96	0.70
*Thermostichus* sp. M44	0.23	0.10	0.46	−0.09	0.08	46.55	62.72	44.48	66.37	0.65
*Thermostichus* sp. MAXBIN	0.22	0.06	0.44	−0.06	0.08	49.44	61.14	43.45	58.87	0.57
Thermostichus sp. Nb3U1	0.22	0.06	0.44	−0.07	0.08	48.46	61.06	43.00	61.00	0.60
*Thermostichus vulcanus* Rupite	0.22	0.06	0.44	−0.09	0.08	48.83	60.65	43.25	60.13	0.59

The CU and CUB indices are shown in Table 2. Similar to the patterns of base composition, the *Pseudocalidococcus* had the lowest CAI value (0.20), while *Thermostichus* had the highest (0.22–0.24). *Parathermosynechococcus* and *Thermosynechococcus* had similar CAI values (0.21–0.22). Notably, *Pseudocalidococcus* had a significantly lower CBI value (−0.05 to −0.04) compared to the other three thermophilic genera (0.00–0.13), indicating different levels of gene expression between thermophiles and non-thermophiles. Similarly, the lowest FOP values (0.38) were observed in *Pseudocalidococcus* and the highest (0.44–0.48) in *Thermostichus*. Furthermore, more than 61% of the genes in each genome showed negative GRAVY values, indicating their hydrophilic character, while the remaining genes could be hydrophobic proteins. Interestingly, the same AROMO values (0.08) were found to be present in all the genomes studied. Additionally, *Thermostichus* had a lower ENC value (47.34 on average) compared to the other three genera (48.35–49.91), showing a different CU preference among the genera. Collectively, these indices reveal different CU patterns especially between thermophilic species and non-thermophilic species.

### Correlation of the CU and CUB indices

**FIGURE 1:**
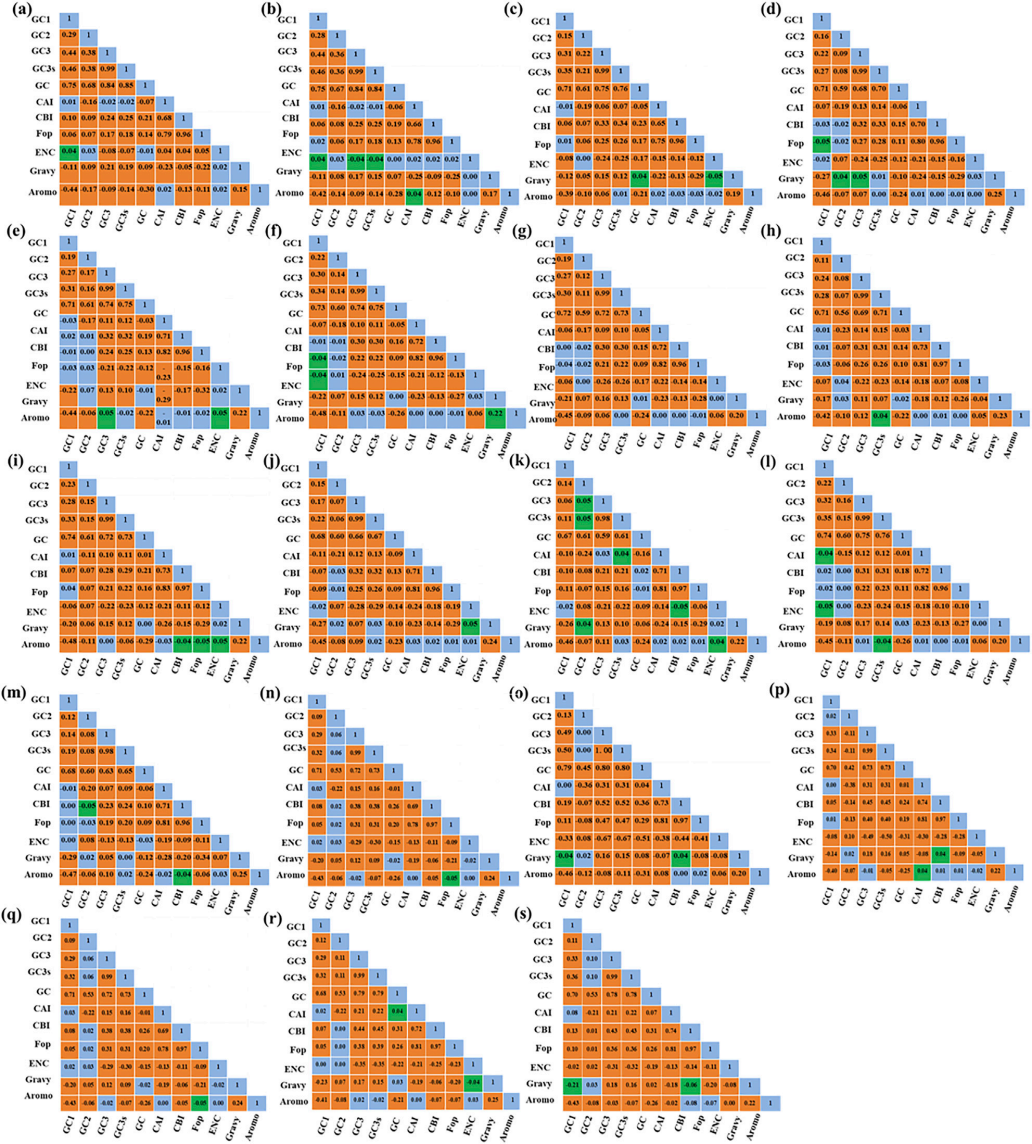
Pearson’s correlation analysis of different CU and CUB indices of the studied cyanobacterial species. Two significance levels were applied, namely 0.05 (green) and 0.01 (orange). **(A)** *Pseudocalidococcus* BACA0444; **(B)** *Pseudocalidococcus* PCC 6312; **(C)** *Parathermosynechococcus* PCC 6715; **(D)** *Thermosynechococcus* NK55; **(E)** *Thermosynechococcus* E542; **(F)** *Thermosynechococcus* CL-1; **(G)** *Thermosynechococcus* TA-1; **(H)** *Thermosynechococcus* BP-1; **(I)** *Thermosynechococcus* HN-54; **(J)** *Thermosynechococcus* KatS; **(K)** *Thermosynechococcus* M55; **(L)** *Thermosynechococcus* PP45; **(M)** *Thermosynechococcus* Uc; **(N)** *Thermostichus* JA-2-3Ba; **(O)** *Thermostichus* JA-3-3Ab; **(P)** *Thermostichus* M44; **(Q)** *Thermostichus* MAXBIN; **(R)** *Thermostichus* Nb3U1; **(S)** *Thermostichus* Rupite.

The results of the correlation analysis of the CU and CUB indices are summarized in Figure 1. In general, GC1 was found to be significantly correlated with GC2, GC3, GC3s and GC content in all four cyanobacterial genera except for *Thermostichus* sp. M44. GC2 was found to be significantly associated with GC3, GC3s and GC in all genera except for four *Thermostichus* genomes, while GC3 was significantly related to GC3s and GC in all four genera. The correlations of the other indices were found to be quite variable among the genomes, indicating that CUB is affected by a variety of factors.

### Neutral analysis

A neutrality plot can clarify the impact of mutational pressure and the role of natural selection on CUB. A regression slope of 0, with no significant correlation between GC12 and GC3, indicates complete dependence on natural selection. Conversely, a slope approaching or equal to 1 with a significant correlation suggests that mutational pressure substantially influences the gene (Sueoka, 1988). As shown in Supplementary Table S1, the regression slopes varied from 0.0966 to 0.8388, with R^2^ values ranging from 0.0053 to 0.2605. Pearson’s correlation analysis identified a significant correlation (*P* < 0.01) between GC12 and GC3 across the 19 genomes examined. The collective data indicates that mutational pressure predominantly influences CUB in *Pseudocalidococcus* and *Parathermosynechococcus*, whereas natural selection is the principal factor driving CUB in *Thermosynechococcus*. In *Thermostichus*, CUB is primarily affected by mutational pressure in JA-2-3Ba, JA-3-3Ab, and Nb3U1, while natural selection is the driving force of CUB in the remaining three genomes. These differing influences may shape the evolutionary trajectories of these cyanobacterial genera.

### Influence of ENC on CUB

The average ENC values of the four cyanobacterial genera ranged from 47.34 to 49.74 and were found to be all higher than 35, indicating that these genera do not have a strong codon usage preference (Supplementary Figure S1). The ENC-GC3s plots show that the genes of these cyanobacterial species deviate significantly from the expected ENC plot curve. Hence, mutational pressure alone does not account for CUB; other factors, such as natural selection, also contribute significantly to CUB formation. The ENC_ratio_ values for these cyanobacteria ranged from 3.18% to 8.49% (Supplementary Figure S2). Furthermore, the ENC values were suggested to be influenced by the GC3s values as per the calculation formula, highlighting the crucial role of GC3s in CUB formation. Thus, the findings confirm that factors beyond mutational pressure, such as natural selection, play a key role in shaping the CUB in these cyanobacterial genera.

### Preference for the third codon position

The PR2-bias plot analysis (Supplementary Figure S3) suggests that the genes of the 19 genomes were not uniformly distributed among the four quadrants. The genes of *Pseudocalidococcus*, *Parathermosynechococcus* and *Thermosynechococcus* were found to be predominantly distributed in the quadrants of G3/(G3 + C3) < 0.5 and A3/(A3 + T3) < 0.5, whereas the genes of *Thermostichus* were found to be predominantly distributed in the quadrants of G3/(G3 + C3) > 0.5 and A3/(A3 + T3) < 0.5. All the results suggest a strong preference for the third base of the codons in these cyanobacterial species. Thus, other factors, such as natural selection, play a key part in the process of CUB in these cyanobacteria.

### Correspondence analysis

RSCU values-based correspondence analysis indicates that the four axes contributed the most to the variance, with average contributions of 14.67%, 10.66%, 10.03%, and 9.67%, respectively (Supplementary Figure S4). Among them, axis 1 contributed the most to the variance. The correlation between axis 1 and GC, GC3s, ENC, CAI, CBI and FOP was further analyzed, and the results show that only in *Thermostichus* (except for *Thermostichus* MAXBIN) axis 1 significantly correlated (*P* < 0.01) with all these investigated indicators. In addition, there were large differences among the RSCU values of genes, suggesting that the synonymous CUs of the genes are differentiated.

### Optimal codon identification

**FIGURE 2:**
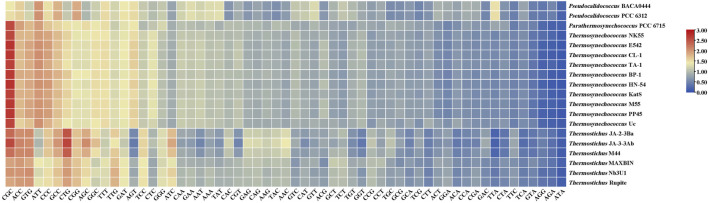
The RSCU values of the genomes of the studied cyanobacterial species. A gradient from blue to red indicates that the average RSCU value of the codon is from low to high.

**FIGURE 3:**
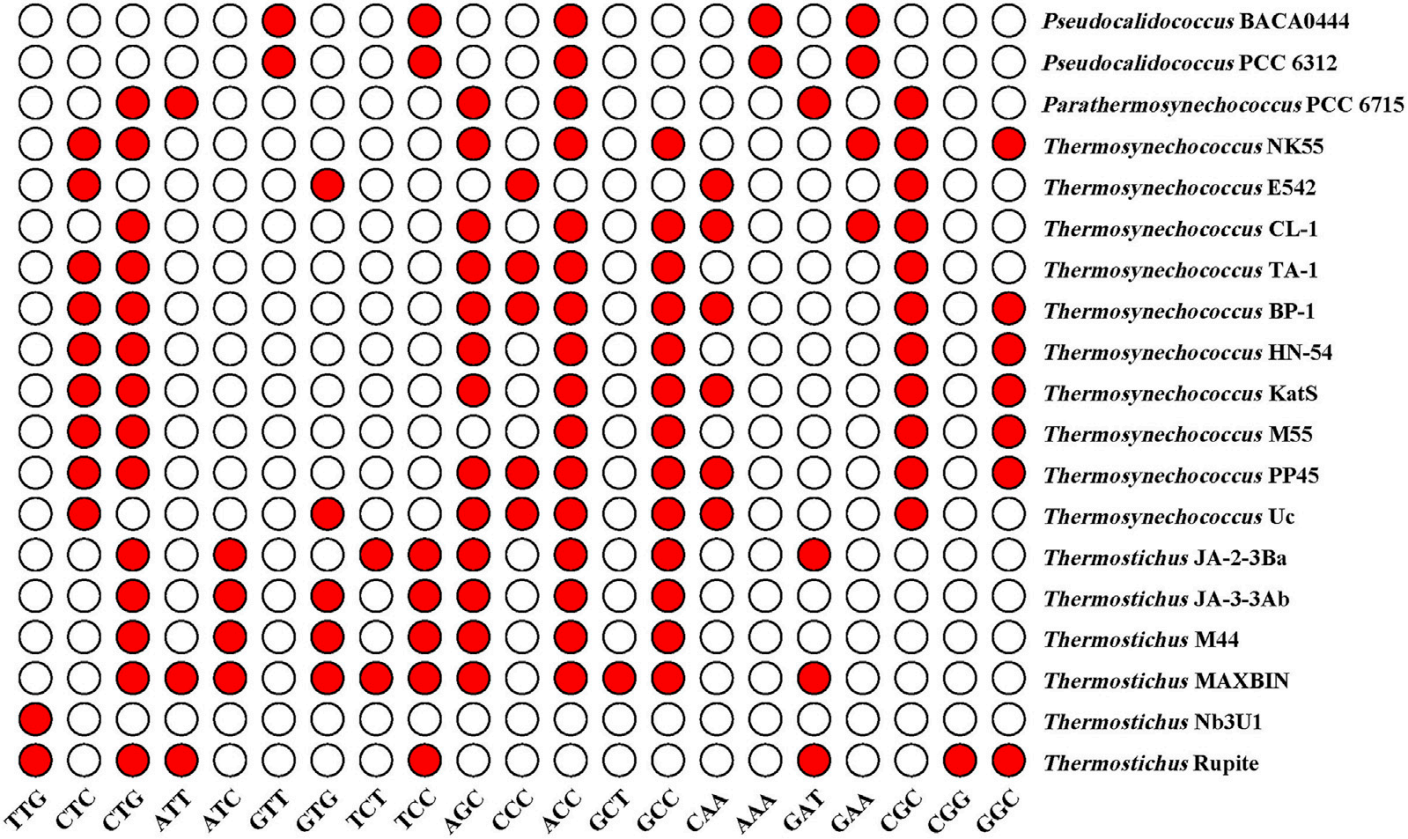
Optimal codons of the studied cyanobacterial species (ΔRSCU >0.08 and RSCU >1).

As shown in Figure 2, the RSCU values for specific codons were found to be different among the genera. The codon CGC encoding arginine (Arg) had a high RSCU value (2.37) and was therefore strongly preferred in the three thermophilic genera. RSCU analysis shows that *Pseudocalidococcus* contained an average of 25 high-frequency codons (RSCU > 1.0), followed by 23 in *Thermosynechococcus*, 22 in *Thermostichus*, and 20 in *Parathermosynechococcus* (Figure 2). In total, 15 high-frequency codons were found to be common to all the studied cyanobacterial genomes. Of these 15 common codons, 10 end with C/G and five end with T/A, suggesting that these common codons tend to end in G/C. Except for *Pseudocalidococcus*, intraspecies variation of optimal codons was evident within the other three thermophilic genera (Figure 3). The optimal codons of the thermophilic genera end mainly in G/C, while A/T is preferred in that of the non-thermophilic genera. Among the optimal codons, ACC was found to be the most frequently used, followed by CTG, AGC, GCC, and CGC. In addition, genus-specific optimal codons were present, such as AAA and GTT in *Pseudocalidococcus*.

### Codon context pattern

The type of codon pair identified was 3,904, 3,900, 3,898, and 3,899 on average in *Pseudocalidococcus*, *Parathermosynechococcus*, *Thermosynechococcus*, and *Thermostichus*, respectively (Supplementary Table S2). The most used codon pair differed among the four genera, namely CAG-GCC in *Pseudocalidococcus*, GAU-CGC in *Parathermosynechococcus* and *Thermosynechococcus*, and CUG-CUG in *Thermostichus*. The top 10 of the most used codon pairs also differed among genera. All codons in each genome were arranged into a 64 × 64 matrix, where each codon was paired with 63 other codons (Supplementary Figure S5). These findings reveal different codon context patterns among these cyanobacterial genera. Moreover, the co-codon context results for the 3′ sequence of the start codon and the 5′ sequence of the stop codon indicate that specific sequences are often used as contexts for the start and stop codons of these cyanobacterial species (Supplementary Table S3). First, GCC or CCC is the most common codon in the 5′-context of stop codons UAA and UAG for the vast majority of the species, while the 5′-codon of the stop codon UGA varies among species. Second, the 5′- context most frequently avoided by stop codons varies considerably among species. Third, GUG and GCC are respectively the most common 3′-contexts of the start codon AUG for *Pseudocalidococcus* and *Thermostichus* and *Parathermosynechococcus* and *Thermosynechococcus*, whereas the UAA and UGA codons are the most frequently avoided 3′-contexts for AUG. All the results suggest a degree of non-random use of start and stop signal codon settings in these cyanobacterial species.

### Phylogenetic relationship

**FIGURE 4:**
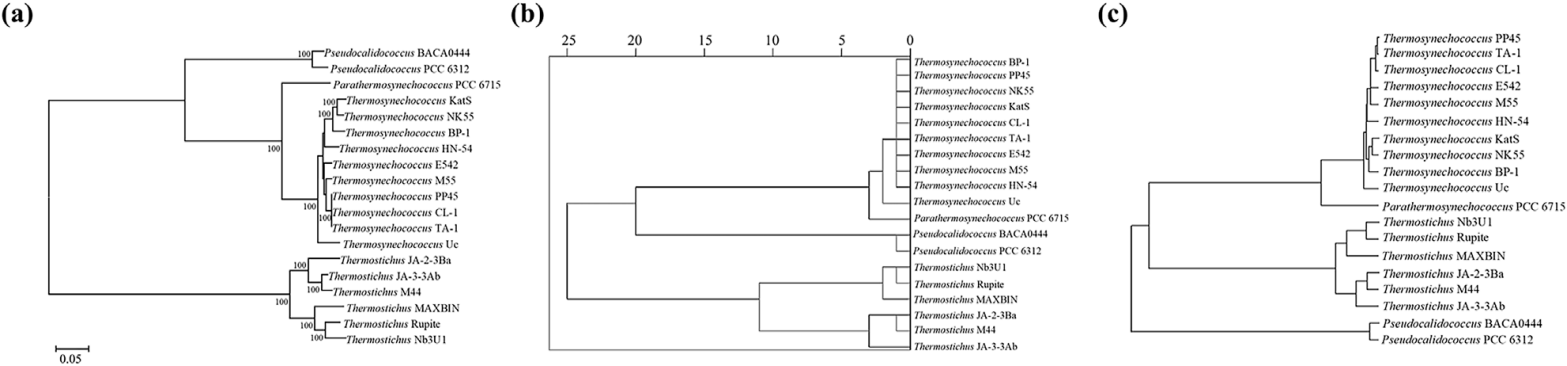
Relationship inferences of the studied cyanobacterial species. **(A)** Phylogenomic tree based on multiple sequence alignment of 120 bacterial marker genes; **(B)** hierarchical clustering generated from the RSCU values; **(C)** clustering inferred from codon context patterns.

The ML phylogenomic tree of the cyanobacterial species (Figure 4A) was found to be similar to the clustering inferred based on the RSCU values (Figure 4B). Both topologies indicate that *Parathermosynechococcus* and *Thermosynechococcus* are more closely related genera, from which *Pseudocalidococcus* was divergent within the family Thermosynechococcaceae. Intriguingly, the clustering inferred by codon context patterns (Figure 4C) was different from the other two phylograms. The *Pseudocalidococcus* is even more divergent from *Parathermosynechococcus* and *Thermosynechococcus* than *Thermostichus*. Thus, codon context patterns may be more related to thermotolerance than to phylogenetic relationships.

## Discussion

Genetic codons are a core component that is associated with genetic material, amino acids, and proteins in organisms. Researching CUB will provide reliable information for the investigation of genetic structure and evolutionary trends, along with protein expression and related functions (Wang et al., 2018; Chakraborty et al., 2020). Numerous biological factors have been proven to affect CUB. Among them, mutation and natural selection are the primary factors influencing CUB (Sundararajan et al., 2016; Meyer, 2021). Recently, the availability of more and more cyanobacterial genomes has facilitated the rapid investigation of the molecular components of various systems and the structural traits from a genome perspective (Tang et al., 2023a; Tang et al., 2023b; Tang et al., 2024a). Nevertheless, the characteristics of CUB for the genomes of thermophilic cyanobacteria and the closely related non-thermophilic cyanobacteria have not been fully understood.

The current study comprehensively analyzed the characteristics and sources of variation of CUB and the phylogenetic relationships of the 19 genomes from four cyanobacterial genera. Nucleotide composition showed distinct patterns between non-thermophiles and thermophiles (Table 2). In addition, the most thermophilic cyanobacterium *Thermostichus* exhibited a different nucleotide composition than the thermophilic cyanobacteria *Parathermosynechococcus* and *Thermosynechococcus*. The latter always showed similar patterns of CU and CUB indices. These findings indicate that the CU and CUB appear to be related to thermotolerance. The genes of the three thermophilic genera tend to end in G/C, while those of *Pseudocalidococcus* usually end in A/T. This observation is consistent with previous findings that codons ending in A and/or T are commonly used in the genomes of marine and freshwater cyanobacteria (Yu et al., 2012) and that higher genomic GC content is important for thermophilic organisms to maintain nucleic acid structure (Musto et al., 2006). Moreover, this suggests that factors in different life modes may influence the pattern of synonymous codon usage in cyanobacterial genomes (Rajneesh et al., 2017).

Significant correlations are present between codon base composition and CAI, CBI and FOP (Figure 1), suggesting that base composition affects CUB. In addition, as shown by the results of RSCU analysis (Figure 2), both high-frequency codons and optimal codons prefer to end in G/C, which further demonstrates the base preference of the surveyed cyanobacterial species at the third position of the codon. The CUB among the four genera are distinct based on the RSCU values suggests that *Parathermosynechococcus* and *Thermosynechococcus* have similar pattern of CUB, which is distinct from that of the other genera. Since the two genera are phylogenetically close (Figure 4A), the data presented here is in line with the proposal that genetically related species usually have very analogous CUB (Sharp et al., 1988). Furthermore, different genera vary in the types of codons identified as both high-frequency codons and optimal codons. Thus, this finding could serve as a benchmark for optimizing codons for heterologous expression.

Regarding the origin of codon variation, the results of the ENC-GC3s and PR2 plot analysis (Supplementary Figures S1, S3) suggest that both mutational pressure and natural selection significantly influence CUB in these cyanobacterial species. The results of neutrality analyses (Supplementary Table S1) suggest that the primary driving force of CUB differs among cyanobacterial species, which may result in different modulation of codon usage for environmental adaptation of microorganisms (Arella et al., 2021). The observed divergence in the driving force within *Thermostichus* may be attributed to niche temperature, which can dramatically vary from 50 to 72°C among *Thermostichus* strains (Pierpont et al., 2024). The strength of the driving force may also impact the strength of the preference (Xu et al., 2024). It is noteworthy that although the methods here have been widely used in CUB studies, they are based on the assumption of strand symmetry in composition and mutation (Sueoka, 1995). Future work could take into account context-dependent mutational dynamics, which use different assumptions to examine how the context-dependence of mutations affects CUB (Morton, 2022).

Codon context refers to the representation of the arrangement of consecutive codon pairs, revealing the preference for these pairs within an organism (Parvathy et al., 2022). This preference may be conserved or genus/species-specific. The codon pair pattern (Supplementary Table S2) is considerably distinguishable among the four genera. Furthermore, specific codon sequences are preferentially used in the 3′ and 5′ contexts of the start codon and the stop codons, respectively (Supplementary Table S3). The biased usage of the sequence context of start and stop codons may affect the initiation and termination of gene translation (Behura and Severson, 2012; Krafczyk et al., 2021). Moreover, mostly homogeneous codon pairs are ubiquitously frequent in these cyanobacterial species (Supplementary Figure S5). From an economic perspective, using a homogeneous codon context during translation may be energetically less expensive than using a context with distinct codon sequences corresponding to ribosomal A and P sites (Behura and Severson, 2012). Interestingly, the codon pair pattern classified *Pseudocalidococcus* as the most divergent group (Figure 4C) from the other three thermophilic genera, which is entirely different from the phylogenomic relationship (Figure 4A). This is consistent with previous observations that phenotypic traits, rather than phylogenetic relatedness, underlie similarities in CUB among organisms (Botzman and Margalit, 2011). Indeed, it has been shown that species with given phenotypic traits and living in similar environmental conditions, e.g., thermal niches versus non-thermal niches here, show similar codon preferences, indicating an evolutionary convergence of CUB and adaptation in groups of organisms sharing similar physiology and/or living in similar habitats (Masłowska-Górnicz et al., 2022). Several processes, e.g., lateral gene transfer, have been suggested to contribute to the convergence of codon adaptation to environmental parameters (like pressure, salinity and temperature) (Carbone et al., 2005).

Noteworthy is the significant difference in genome size between thermophiles and non-thermophiles or among thermophiles (Table 1). However, genome size is considered to either play a subsidiary role or to rely indirectly on different mutator genes to fine-tune the GC content (Wu et al., 2012). Thus, it is difficult to predict *a priori* selection on CUB based on genome size (LaBella et al., 2019). In addition, although CU can vary not only between organisms but also within different regions of a genome and even within a gene (Hooper and Berg, 2000), it is known that a specific codon usage characterizes each bacterial species and that the majority of its genes share such a bias (Plotkin and Kudla, 2011), indicating that the whole genome, rather than individual genes, is the unit of selection. Moreover, it has been suggested that codon bias in specific categories of genes is a re-modulation of the different CUB of the species (Dilucca et al., 2015).

In conclusion, this study revealed that CUB is a crucial characteristic of genome evolution in cyanobacterial species. The information obtained from this study may be helpful for a better understanding of translational selection between non-thermophiles and thermophiles and between Thermosynechococcaceae and Thermostichaceae. Moreover, transgenic technology is expected in the future to explore genes with underlying important traits in Thermosynechococcaceae and Thermostichaceae species, further facilitating in-depth studies on the biotechnological potentials of these cyanobacterial species.

## Data Availability

The data presented in this study are openly available in the National Center for Biotechnology Information https://www.ncbi.nlm.nih.gov/genome/.

## Footnotes

1
https://codonw.sourceforge.net/
