# Supplementary material for: Comparative genomics of thermosynechococcaceae and thermostichaceae: insights into codon usage bias
Source: Acta Biochim Pol. 2025 Jan 8;71:13825. doi: 10.3389/abp.2024.13825 (PMC11750575; doi:10.3389/abp.2024.13825)

**Supplementary Figure 1** ENC-GC3 analyses of the genes of the cyanobacterial species studied. (a) *Pseudocalidococcus* BACA0444; (b) *Pseudocalidococcus* PCC 6312; (c) *Parathermosynechococcus* PCC 6715; (d) *Thermosynechococcus* NK55; (e) *Thermosynechococcus* E542; (f) *Thermosynechococcus* CL-1; (g) *Thermosynechococcus* TA-1; (h) *Thermosynechococcus* BP-1; (i) *Thermosynechococcus* HN-54;（j）*Thermosynechococcus* KatS; (k) *Thermosynechococcus* M55; (l) *Thermosynechococcus* PP45; (m) *Thermosynechococcus* Uc; (n) *Thermostichus* JA-2-3Ba; (o) *Thermostichus* JA-3-3Ab; (p) *Thermostichus* M44; (q) *Thermostichus* MAXBIN; (r) *Thermostichus* Nb3U1; (s) *Thermostichus* Rupite.


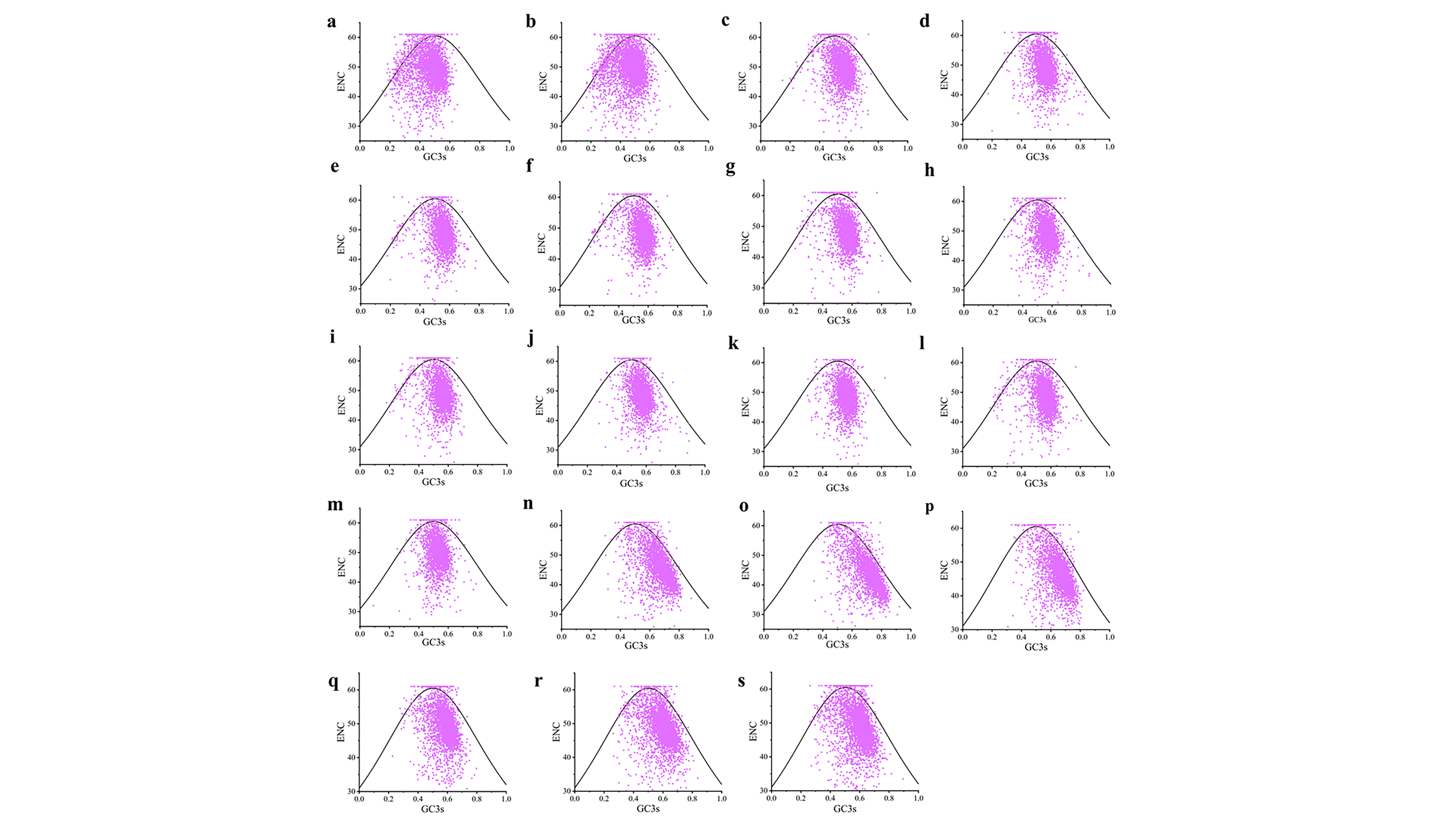


**Supplementary Figure 2** ENC_Ratio_ values in the cyanobacterial species studied.**
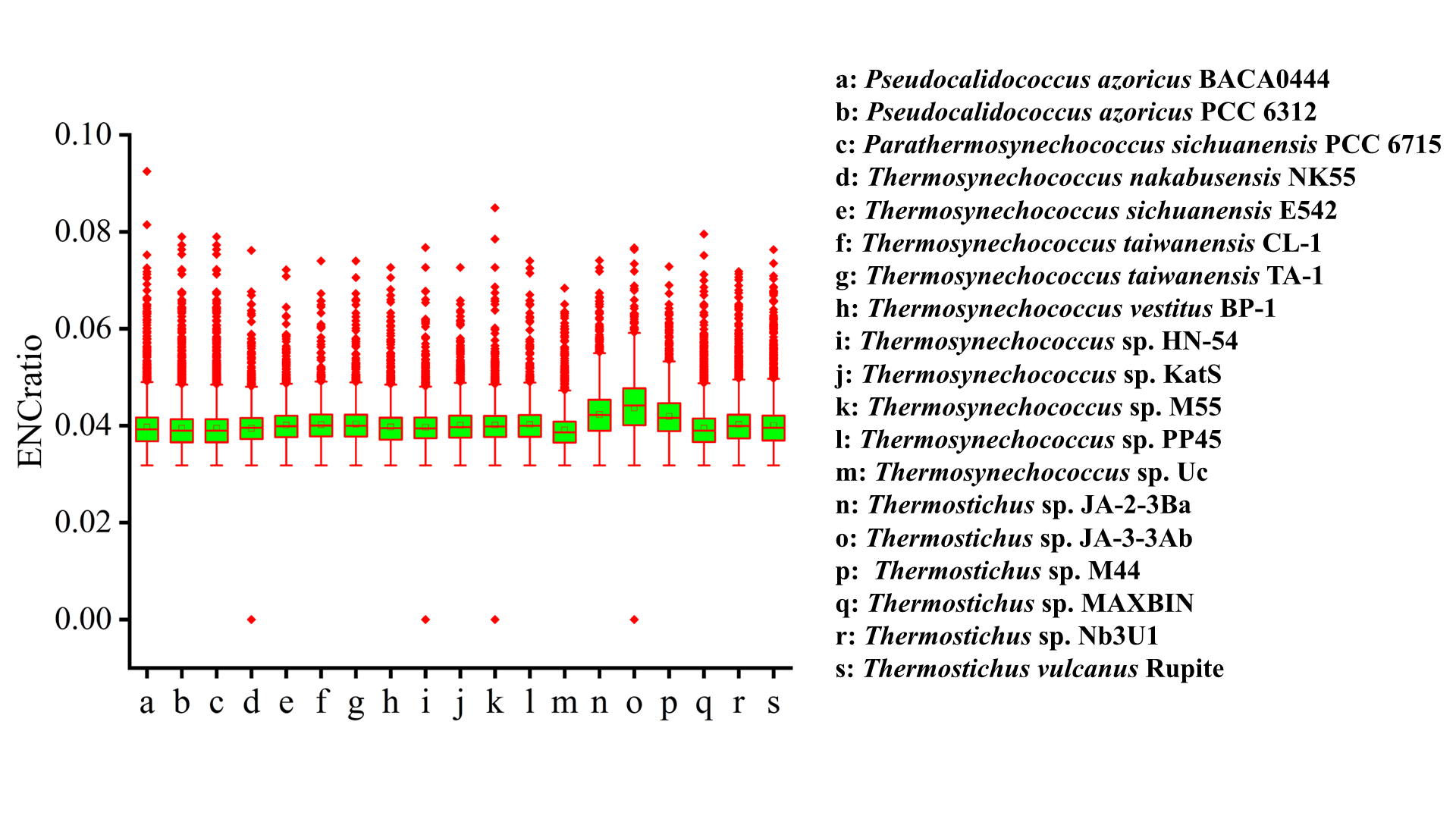
**

**Supplementary Figure 3** Parity Rule 2 (PR2) analyses of the genes of the cyanobacterial species studied. (a) *Pseudocalidococcus* BACA0444; (b) *Pseudocalidococcus* PCC 6312; (c) *Parathermosynechococcus* PCC 6715; (d) *Thermosynechococcus* NK55; (e) *Thermosynechococcus* E542; (f) *Thermosynechococcus* CL-1; (g) *Thermosynechococcus* TA-1; (h) *Thermosynechococcus* BP-1; (i) *Thermosynechococcus* HN-54;（j）*Thermosynechococcus* KatS; (k) *Thermosynechococcus* M55; (l) *Thermosynechococcus* PP45; (m) *Thermosynechococcus* Uc; (n) *Thermostichus* JA-2-3Ba; (o) *Thermostichus* JA-3-3Ab; (p) *Thermostichus* M44; (q) *Thermostichus* MAXBIN; (r) *Thermostichus* Nb3U1; (s) *Thermostichus* Rupite.


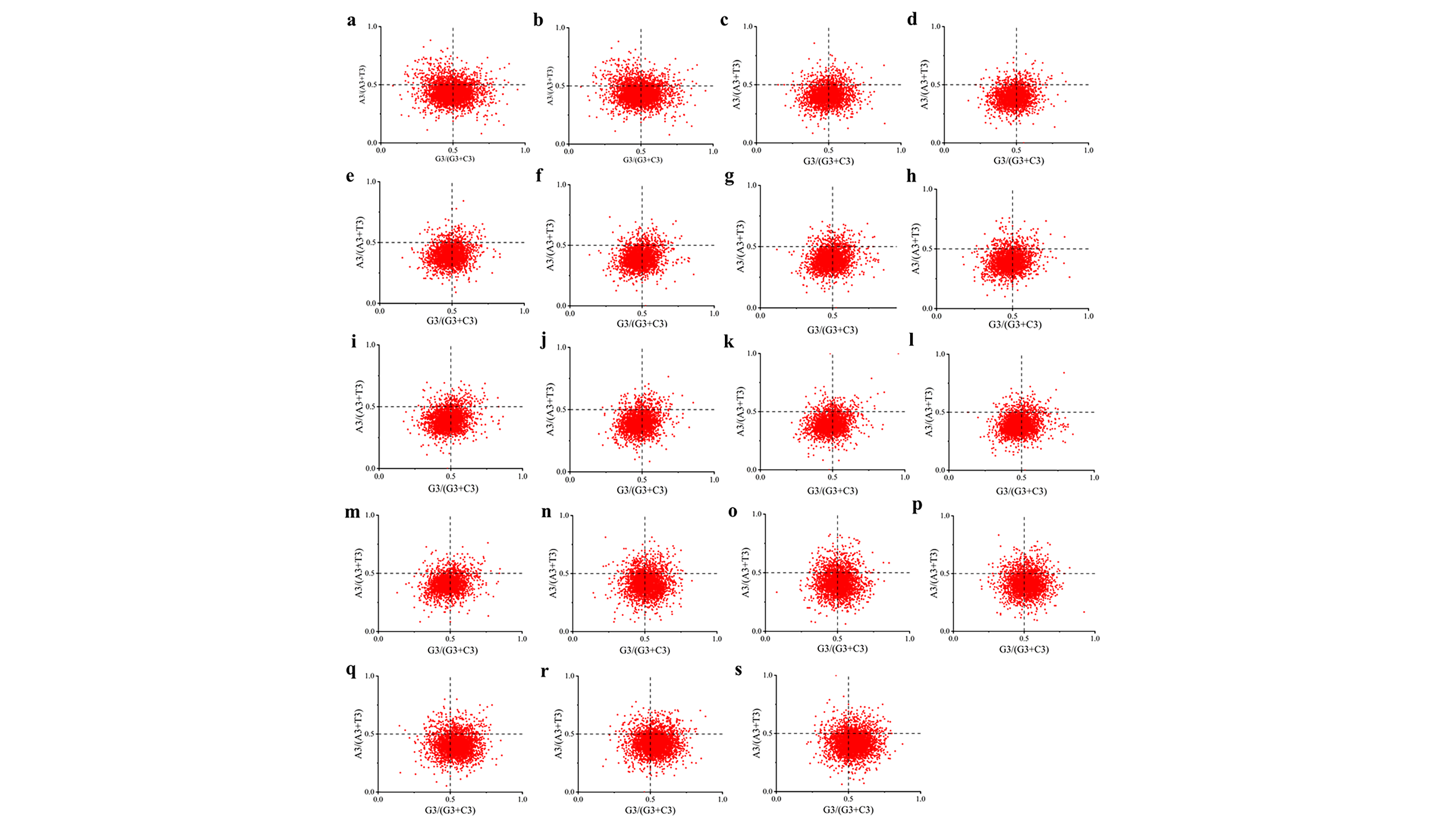


**Supplementary Figure 4** COA analyses of the genes of the cyanobacterial species studied. (a) *Pseudocalidococcus* BACA0444; (b) *Pseudocalidococcus* PCC 6312; (c) *Parathermosynechococcus* PCC 6715; (d) *Thermosynechococcus* NK55; (e) *Thermosynechococcus* E542; (f) *Thermosynechococcus* CL-1; (g) *Thermosynechococcus* TA-1; (h) *Thermosynechococcus* BP-1; (i) *Thermosynechococcus* HN-54;（j）*Thermosynechococcus* KatS; (k) *Thermosynechococcus* M55; (l) *Thermosynechococcus* PP45; (m) *Thermosynechococcus* Uc; (n) *Thermostichus* JA-2-3Ba; (o) *Thermostichus* JA-3-3Ab; (p) *Thermostichus* M44; (q) *Thermostichus* MAXBIN; (r) *Thermostichus* Nb3U1; (s) *Thermostichus* Rupite.


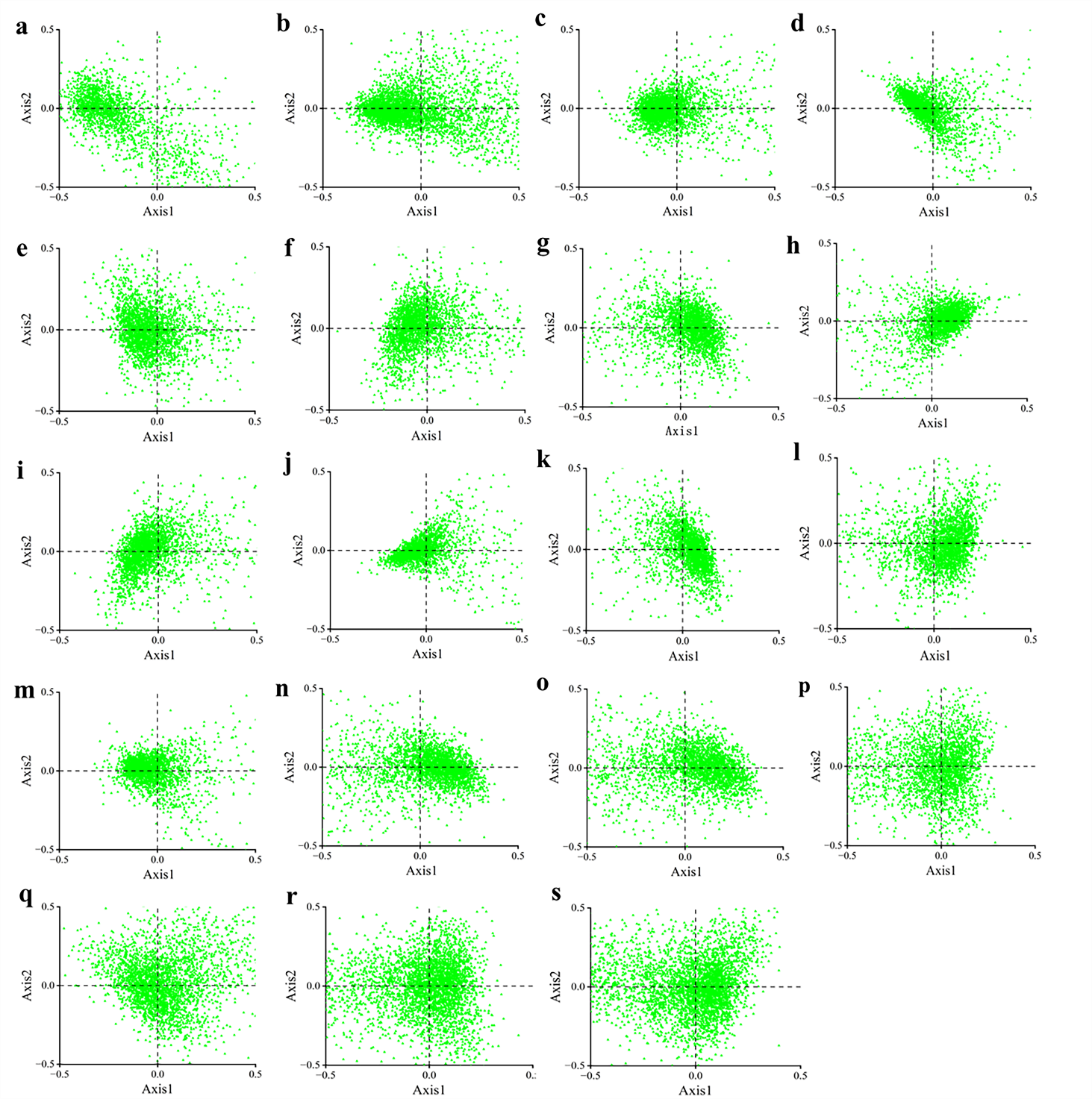


**Supplementary Figure 5** Codon context pattern of the cyanobacterial species studied. (a) *Pseudocalidococcus* BACA0444; (b) *Pseudocalidococcus* PCC 6312; (c) *Parathermosynechococcus* PCC 6715; (d) *Thermosynechococcus* NK55; (e) *Thermosynechococcus* E542; (f) *Thermosynechococcus* CL-1; (g) *Thermosynechococcus* TA-1; (h) *Thermosynechococcus* BP-1; (i) *Thermosynechococcus* HN-54;（j）*Thermosynechococcus* KatS; (k) *Thermosynechococcus* M55; (l) *Thermosynechococcus* PP45; (m) *Thermosynechococcus* Uc; (n) *Thermostichus* JA-2-3Ba; (o) *Thermostichus* JA-3-3Ab; (p) *Thermostichus* M44; (q) *Thermostichus* MAXBIN; (r) *Thermostichus* Nb3U1; (s) *Thermostichus* Rupite.


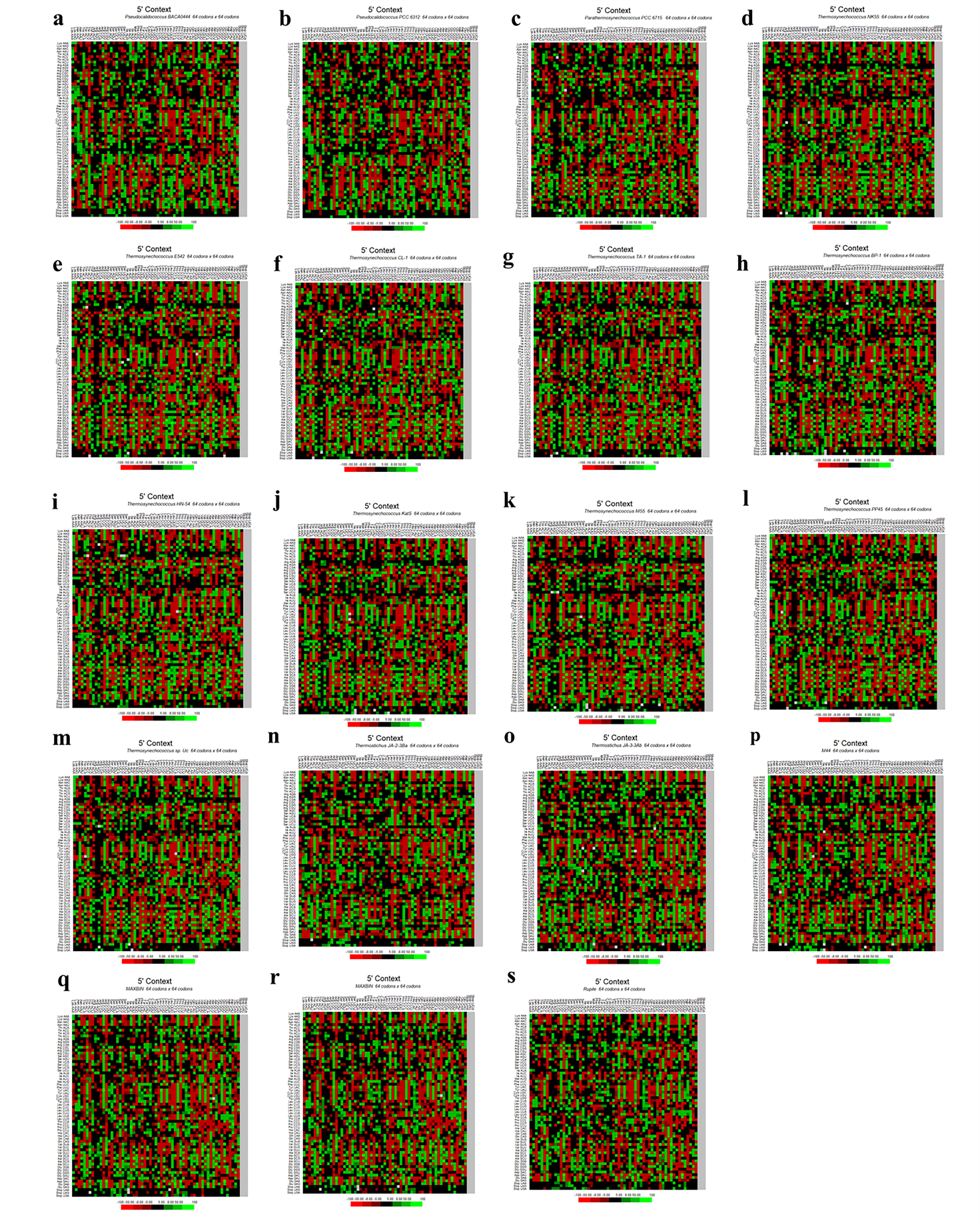

Supplement: Supplementary file 1 [file DataSheet1.docx]
